# Leadership, action, learning and accountability to deliver quality care for women, newborns and children

**DOI:** 10.2471/BLT.17.197939

**Published:** 2018-02-05

**Authors:** Abosede Adeniran, Andrew Likaka, Anneka Knutsson, Anthony Costello, Bernadette Daelmans, Blerta Maliqi, Daniel Burssa, Joseph Freer, Ian Askew, Lisa Bowen, Lily Kak, Lori McDougall, Nabila Zaka, Özge Tunçalp, Petra Tenhoope-Bender, Shamsuzzoha Babar Syed, Stefan Swartling Peterson, Thiago Luchesi, Willibald Zeck, Wilson Were,, Pierre Barker, Zainab Naimy

**Affiliations:** a Child Health Division, Federal Ministry of Health, Abuja, Nigeria.; b Ministry of Health Malawi, Lilongwe, Malawi.; c United Nations Population Fund, New York, United Sates of America (USA).; d Family, Women’s and Children’s Health, World Health Organization, avenue Appia 20, 1211 Geneva 27, Switzerland.; e Federal Ministry of Health, Addis Ababa, Ethiopia.; f White Ribbon Alliance, Washington, USA.; g United States Agency for International Development, Washington DC, USA.; h The Partnership for Maternal, Newborn & Child Health, World Health Organization, Geneva, Switzerland.; i United Nations Children's Fund, New York, USA.; j Department of Service Delivery and Safety, World Health Organization, Geneva, Switzerland.; k Save the Children, Geneva, Switzerland.; l Institute for Healthcare Improvement, Cambridge, USA.

The Member States of the World Health Organization (WHO) are committed to achieve quality, equity and dignity for women, newborns and children as reflected in the four World Health Assembly Resolutions.^1^^–^^4^ These resolutions provide the foundation to reach the targets for maternal, newborn and child health and survival^5^ of the sustainable development goal on health, and universal health coverage. Although coverage of health services has increased, many women, newborns and children continue to die from poor care practices, even after reaching a health facility.^6^^,^^7^ Poor care practices are not limited to the medical aspects of care or resources needed to provide this care; research has demonstrated a disrespectful or neglectful treatment in the facilities that negatively impacts the care outcomes for women and newborns.^8^ Implementing an approach to improve quality of care at scale that is effective and sustainable is critical to further reduce mortality and improve health outcomes.^9^

Recognizing the need for action, the national governments of Bangladesh, Côte d’Ivoire, Ethiopia, Ghana, India, Malawi, Nigeria, Uganda and United Republic of Tanzania, together with WHO, the United Nations Children’s Fund (UNICEF), the United Nations Population Fund (UNFPA), implementation partners and other stakeholders, have established the Network for Improving Quality of Care for Maternal Newborn and Child Health care.^10^ The network has agreed to pursue the ambitious goals of halving maternal and newborn deaths and stillbirths and improving experience of care in participating health facilities within five years of implementation. Under the leadership of the participating countries’ health ministries, the network will support the implementation of national frameworks for quality improvement by pursuing four strategic objectives: (i) leadership by building and strengthening national institutions and processes for improving quality of care; (ii) action by accelerating and sustaining implementation of quality-of-care improvement packages through operationalizing a standards-based approach to quality improvement; (iii) learning by promoting joint learning and generating evidence on quality planning, improvement and control of health services; and (iv) accountability by developing, strengthening and sustaining institutions and mechanisms for accountability of quality maternal, neonatal and child health services that are equitable and dignified.

The network’s strategic objectives reflect the necessity expressed by countries and partners to address quality improvement as part of the unfinished survival agenda stated in the *Global strategy for women’s, children’s and adolescents’ health (2016–2030)*^11^ and its related maternal and newborn health action plans.^12^^,^^13^ These objectives also reflect the need to find transformative responses that will support quality improvement in a sustainable way and at scale.^14^

The stakeholders of the network believe that harmonized and coordinated actions to improve quality of care can make a substantial difference and, with the right investment and focus, can drive progress towards the network’s ambitious goals.^15^ In all countries participating in the network, investments in improving the quality of care for mothers, newborns and children have often been project-based and fragmented. As a result, these efforts have not been successfully sustained or scaled up. To address this gap, participating countries have developed and are strengthening their national quality of care policies, strategies and institutions for better quality planning, assurance and improvement. Furthermore, participating governments and implementing partners, through the countries’ strategic coordinating committees, have joined forces to align and harmonize their quality improvement actions, including for maternal and newborn services provided in health facilities.

Actions to improve quality of care have to be informed by evidence. The WHO document *Standards for improving quality of maternal and newborn care in health facilities*^16^ provide the basis for informing these actions. Under the framework of health systems strengthening and with a strong focus on community engagement and accountability, the standards place a strong emphasis on the provision of care that is safe, effective, timely, efficient, equitable and people centred. The standards also underline the experience of care as an integral component of quality.

Health systems are complex and solutions that lead to quality improvement are often context-specific. Much is yet to be learnt and understood about how to effectively deploy evidence-based practice at facility, regional or national levels. Recognizing this complexity and the importance of innovative thinking, the network has prioritized learning as a mechanism that would generate knowledge to carry out sustainable improvements. Informed by implementation needs, the network is bringing together a community of health practitioners from facility, district, national and global levels, who will share implementation ideas and experiences and develop evidence-based yet context-specific strategies for quality improvement. Working through national organizations and institutions, the network aims to become a platform for cross-country and cross-sectoral learning and knowledge exchange. These efforts are in line with the set of evidence-based implementation interventions identified by WHO that can further guide the implementation of improvement agendas.^17^

Measuring progress and demonstrating accountability are crucial mechanisms to support continuous quality improvement. The network’s monitoring working group will closely follow up and account for progress to demonstrate changes in maternal and newborn health outcomes, experience of care and readiness of systems to deliver quality care. Much has been achieved in clarifying relevant indicators of quality and benchmarks for maternal and newborn health,^18^^,^^19^ yet more work is required.^20^ Similarly, more work will be needed to ensure the engagement of women, families, communities and other stakeholders in developing effective accountability mechanisms and using them to achieve quality-of-care outcomes.

Furthermore, the network needs to estimate the cost of providing quality care for maternal and newborn health. In the past decade, countries have successfully invested in developing and costing national health strategies and plans to guide the scaling up of lifesaving interventions. However, quality assurance and quality improvement are often neglected and do not appear among the costed priorities. The network’s countries will identify and cost their quality improvement packages and support systems to respond to this gap.

The network’s structure reflects its most valuable asset: the wealth of knowledge and commitment of all stakeholders who are engaging with countries to identify and implement solutions to improve quality of care and monitor results. The network is deliberately attempting to build a broad-based coalition at regional, national and global levels that is focused on implementation. Each country in the network has brought together partners working on quality improvement and is now focusing on identifying the actions that can be scaled up across their respective systems. The network is implementing the principles of quality, equity and dignity, the three pillars of the Every Woman Every Child global movement.^21^ Working through existing mechanisms such as the Quality, Equity and Dignity Advocacy Working Group, coordinated by the Partnership for Maternal, Newborn & Child Health,^22^ the network supports the broader efforts of the Every Woman Every Child movement and directly contributes towards the *Global strategy for women’s, children’s and adolescents’ health (2016–2030).*

The network provides an opportunity to apply the newest evidence on quality improvement, including the recommendations of the *Lancet global health* Commission on High Quality Health Systems.^23^ At the same time, lessons from in-country implementation will continue to enrich the global evidence and knowledge base.^24^

The network is initially focusing on mothers and newborns but will quickly expand to include child health, and aims to gradually cover the full continuum of care. While the network’s nine countries have taken an active lead, all countries and stakeholders, such as United Nations partners, development agencies, donors, professional associations and civil society, are invited to share their experiences and lessons learnt and to use the network’s knowledge and tools to strengthen quality of care in their respective contexts.
